# Bayesian Random Tomography of Particle Systems

**DOI:** 10.3389/fmolb.2021.658269

**Published:** 2021-05-21

**Authors:** Nima Vakili, Michael Habeck

**Affiliations:** ^1^ Microscopic Image Analysis Group, Jena University Hospital, Jena, Germany; ^2^ Statistical Inverse Problems in Biophysics, Max Planck Institute for Biophysical Chemistry, Göttingen, Germany

**Keywords:** 3D Reconstruction, random tomography, cryo-EM, bayesian inference, coarse-grained modeling, markov chain Monte Carlo, inferential structure determination

## Abstract

Random tomography is a common problem in imaging science and refers to the task of reconstructing a three-dimensional volume from two-dimensional projection images acquired in unknown random directions. We present a Bayesian approach to random tomography. At the center of our approach is a meshless representation of the unknown volume as a mixture of spherical Gaussians. Each Gaussian can be interpreted as a particle such that the unknown volume is represented by a particle cloud. The particle representation allows us to speed up the computation of projection images and to represent a large variety of structures accurately and efficiently. We develop Markov chain Monte Carlo algorithms to infer the particle positions as well as the unknown orientations. Posterior sampling is challenging due to the high dimensionality and multimodality of the posterior distribution. We tackle these challenges by using Hamiltonian Monte Carlo and a global rotational sampling strategy. We test the approach on various simulated and real datasets.

## 1 Introduction

Many different imaging techniques acquire two-dimensional (2D) projection data of an unknown three-dimensional (3D) object. If the projection directions are known, tomographic reconstruction methods can be used to recover the 3D structure of the object (Natterer, 2001). An additional complication arises, if the projection directions are unknown. This imaging modality is of particular relevance to single-particle cryo-electron microscopy (cryo-EM). In recent years, cryo-EM has emerged as a powerful technique to determine the structure of large biomolecular assemblies at near atomic resolution (Frank, 2006). In cryo-EM, many copies of the particle of interest are first applied to a carbon grid and then plunge-frozen to prevent the formation of ice crystals. The frozen randomly orientated particles are imaged with electrons resulting in thousands to millions of noisy projection images. Similar reconstruction problems arise in cryo-electron tomography as well as single-particle diffraction experiments at free-electron lasers (von Ardenne et al., 2018). A completely different field of application is *in situ* microscopy of various specimens such as mesoscopic organisms (Levis et al., 2018).

The reconstruction problem common to all of these imaging methods is to recover a 3D volume from 2D images acquired in random projection directions and has been termed random tomography (Panaretos, 2009). Since the projection directions are unknown, we have to estimate them in the course of the reconstruction. Moreover, to avoid model bias, the desired reconstruction method should not rely on an initial guess of the volume (ab initio reconstruction).

Various ab initio reconstruction methods have been proposed (Bendory et al., 2020) including maximum likelihood via expectation maximization (Scheres et al., 2007) and maximum a posteriori (MAP) estimation (Jaitly et al., 2010; Scheres, 2010, 2012a), regularized maximum likelihood (Scheres, 2012b), stochastic gradient descent (Punjani et al., 2017), common lines (Vainshtein and Goncharov, 1986; Van Heel, 1987; Penczek et al., 1996; Elmlund et al., 2008; Singer and Shkolnisky, 2011; Elmlund and Elmlund, 2012; Lyumkis et al., 2013), the method of moments (Kam, 1980; Levin et al., 2018), random-model methods (Yan et al., 2007; Sanz-Garcia et al., 2010), methods using stochastic hill climbing (Elmlund et al., 2013) or nonlinear dimensionality reduction (Vargas et al., 2014) and frequency marching (Barnett et al., 2017).

These approaches typically reconstruct the unknown volume by solving an optimization problem. However, optimization approaches do not offer any uncertainty quantification. Another drawback is that many reconstruction algorithms are iterative procedures that critically depend on the initialization, which counteracts the idea of achieving an unbiased ab initio reconstruction. Moreover, most algorithms employ a number of ad hoc parameters that need to be tuned by the user and impact the final result in a way that is not always obvious.

Our goal is to develop a fully Bayesian approach to 3D reconstruction using a meaningful model of the unknown structure (including a physically realistic prior) and utilizing sampling algorithms for parameter estimation and uncertainty quantification. In our previous work (Joubert and Habeck, 2015), we already took the first step towards this goal. We considered the reconstruction problem in random tomography as a density estimation problem utilizing a mixture of Gaussians. With the help of conjugate priors and the introduction of latent assignment variables, we could derive analytical updates for a Gibbs sampler that infers the unknown rotations and component means.

However, there are various problems with our previous Gibbs sampling approach. First, Gibbs sampling suffers from slow convergence and depends strongly on the initial conditions. Therefore, to locate the posterior mode many restarts of the Gibbs sampler from varying initial conditions are necessary. Second, our Gibbs sampling algorithm is restricted to a Poissonian likelihood. The Poisson model is limited in that it ignores the effect of the point spread function and correlations in the noise. Third, the prior over the component means (particle positions) is chosen to be a conjugate, zero-centered Gaussian distribution, which is not realistic for biomolecular structures, because it ignores excluded-volume effects.

Here, we overcome these limitations by developing a more general probabilistic model for particle systems and their projection images. We no longer aim to develop analytical updates for the Gibbs sampler, but use of Markov chain Monte Carlo (MCMC) algorithms to infer both the particle positions as well as the unknown rotations. Sampling conformations of the particle system for fixed rotations can be achieved with Hamiltonian Monte Carlo (HMC). To sample the rotations, we use a Metropolis-Hastings algorithm that explores the unit quaternions parameterizing the unknown projection directions. Since Metropolis-Hastings samples a probability distribution only locally, we occasionally run a global sampling step that is computationally more expensive. Using simulated and real experimental data, we demonstrate that our Bayesian approach to random tomography is capable of estimating physically plausible coarse-grained models.

## 2 Probabilistic Model and Posterior Sampling

We aim to reconstruct a 3D volume f(r) for $r\in {\mathbb{R}}^{3}$ and $f:{\mathbb{R}}^{3}\mapsto {\mathbb{R}}_{+}$. We do not observe f(r) directly but only projection images

$$g\left(u\right)=\int^{\text{​}}f\left(R^{T}r\right)\;\text{d}z=\int^{\text{​}}f\left(\theta^{⊥}u+\theta z\right)\;\text{d}z=:X_{\theta }\left[f\right]\left(u\right)$$ (1)

where R∈SO(3) is a 3D rotation matrix whose last row $\theta \in {\mathbb{R}}^{3}$ is a unit vector pointing into the projection direction, and $\theta^{⊥}\in {\mathbb{R}}^{3\times 2}$ is the matrix whose columns span the plane orthogonal to θ such that $R^{T}=\left[\theta^{⊥},\theta \right]$. Throughout this article, $u\in {\mathbb{R}}^{2}$ denotes a position in the projection image, and $r\in {\mathbb{R}}^{3}$ a position in the volume. The integral transform $X_{R}\left[f\right]$ (Eq. 1) is known as the X-ray transform or John transform (Natterer, 2001). In 2D, the X-ray transform is identical to the Radon transform. The reconstruction problem in random tomography is to estimate f(r) from *N* random projection directions $\theta_{n}$, or equivalently $R_{n}$, such that

$$g_{n}\left(u\right)=X_{\theta_{n}}\left[f\right]\left(u\right)+n\left(u\right),\;n=1,\ldots ,N$$ (2)

where n(u) is the noise.

### 2.1 Kernel Expansion of Images and Volumes

The standard discretization of images and volumes is based on pixels and voxels placed on regular 2D and 3D grids. Instead, we expand images and volumes into sums of basis functions that can be centered at irregular positions (as in meshless methods). We use a radial basis function (RBF) kernel *ϕ* such that the kernel expansion of the volume becomes

$$f\left(r\right)=\overset{K}{\underset{k=1}{\sum }}w_{k}\;\phi \left(r-x_{k}\right)$$ (3)

where *K* is the number of basis functions, ∥⋅∥ is the Euclidean norm, $w_{k}$ a coefficient or weight (if $w_{k}>0$) and $x_{k}\in {\mathbb{R}}^{3}$ a position vector that determines the center of the *k*th kernel. We can represent members of a reproducing kernel Hilbert space using this expansion. RBF representations are widely used in machine learning (Schölkopf and Smola, 2002), image processing (Takeda et al., 2007) and numerical applications (Schaback and Wendland, 2006).

**FIGURE 1:**
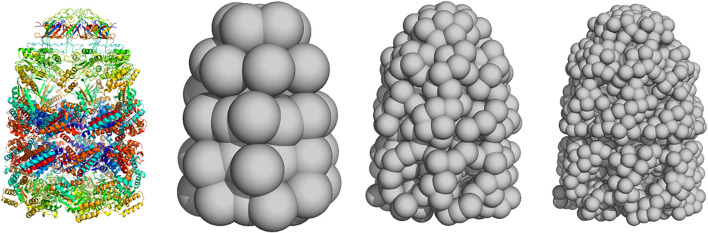
Coarse-grained representation of GroEL/GroES using a varying number of particles **(left)** atomic structure (PDB code 1aon). Panels **(middle left)** to **(right)** show coarse-grained models using K=50,300, and  1000 particles.

A physical interpretation of the kernel representation is that we model the object as a collection of *K* particles at positions $x_{k}$ with mass $w_{k}>0$. The model (3) can then be interpreted as the blurred version of a particle system:

$$f\left(r\right)=\left(\phi *\overset{K}{\underset{k=1}{\sum }}w_{k}\delta_{x_{k}}\right)\left(r\right)$$ (4)

where $\delta_{x_{k}}$ is the delta function centered a $x_{k}$ and the particle density, $\underset{k}{\sum }w_{k}\delta_{x_{k}}$, is blurred by a convolution (denoted by ∗) with the RBF kernel. The particle locations and weights $\left\{\left(x_{k},w_{k}\right);k=1,\ldots ,K\right\}$ can also be viewed as a weighted point cloud. The component means $x_{k}$ could be fixed to a regular 3D grid. But we will consider particle systems that are not tied to a grid and can be distributed in an irregular fashion (similar to meshless or meshfree methods used in numerical analysis). Typically, the particle system is a coarse-grained representation of the unknown structure rather than an atomic-resolution representation. Therefore, 3D reconstruction from 2D projection data provides a pseudo-atomic representation whose resolution depends on the number of particles *K* (Figure 1 for an illustration).

One motivation for our choice of the volume representation (Eq. 3) are its efficient transformation properties. Rigid transformations of f(r) involve a shift by the translation vector t and a reorientation brought about by the rotation matrix R. Under the RBF expansion these transformations reduce to rigid transformations of the particle positions:

$$f\left(r\right)\overset{R,t}{\mapsto }f\left(R^{T}\left(r-t\right)\right)=\underset{k}{\sum }w_{k}\;\phi \left(r-Rx_{k}-t\right)=\underset{k}{\sum }w_{k}\;\phi \left(r-x_{k}\text{′}\right)$$ (5)

where $x_{k}\text{′}=Rx_{k}+t$.

There are many options for ϕ(r). We will restrict ourselves to Gaussian RBF kernels. The *d*-dimensional spherical Gaussian is defined by

$$\phi_{d}\left(r;x,\sigma^{2}\right):=\frac{1}{{\left(2\pi \sigma^{2}\right)}^{d/2}}\text{exp}\left\{-\frac{1}{2\sigma^{2}}\|r-x{\|}^{2}\right\}$$ (6)

where σ>0 is the bandwidth of the kernel. The volume representation that we will use throughout this paper is a mixture of *K* spherical Gaussians:

$$f\left(r\right)=\overset{K}{\underset{k=1}{\sum }}w_{k}\;\phi_{3}\left(r;x_{k},\sigma^{2}\right)$$ (7)

This representation is very common in statistics, in particular in density estimation where $x_{k}$ are observed samples resulting in a kernel density estimate of an unknown probability density function. Indeed, our original motivation (Joubert and Habeck, 2015) to choose this representation of f(r) was mainly driven by viewing 3D reconstruction from random projections as an instance of a density estimation problem. Other examples for uses of (Gaussian) particle representations in cryo-EM data analysis such as denoising or the analysis of continuous conformational changes have been proposed by Jin et al. (2014); Jonić et al. (2016); Jonić and Sorzano (2016).

A convenient property of the spherical Gaussian kernel is its behavior under the X-ray transform (Eq. 1):

$$X_{\theta }\left[\phi_{d}\right]\left(u\right)=\int^{\text{​}}\phi_{d}\left(\theta^{⊥}u+\theta z;x,\sigma^{2}\;\right)\;\text{d}z=\phi_{d-1}\left(u;\mathrm{PRx},\sigma^{2}\right)$$ (8)

where again $R={\left[\theta^{⊥},\theta \right]}^{T}\in SO\left(3\right)$ and the 2×3 projection matrix P is

$$P=\left(\begin{array}{ccc} 1 & 0 & 0\\ 0 & 1 & 0 \end{array}\right)$$ (9)

Spherical Gaussians are closed under the X-ray transform, and the projected volume (7) is again a *K* component mixture of spherical Gaussians

$$X_{\theta }\left[f\right]\left(u\right)=\overset{K}{\underset{k=1}{\sum }}w_{k}\;\phi_{2}\left(u;\mathrm{PR}x_{k},\sigma^{2}\right)$$ (10)

with centers $x_{k}\text{′}=\mathrm{PR}x_{k}\in {\mathbb{R}}^{2}$. This fact motivates us to also represent the input images as mixtures of spherical Gaussians in 2D (see *Representation of Projection Images by Point Clouds* for a concrete application).

### 2.2 Probabilistic Model

The unknown parameters of our model are the particle positions $x_{k}$ and weights $w_{k}$ as well as the unknown rotation matrices $R_{n}$. Since we interpret the Gaussian components as particles of equal mass, we fix the weights: $w_{k}=K^{-1}$, such that the main inference parameters are $x_{k}$ and $R_{n}$.

#### 2.2.1 Likelihoods

We tested two probabilistic models for the input data. The first model uses the input images $\left\{g_{n};n=1,\ldots ,N\right\}$ directly. For each image, the intensities are $g_{nm}=g_{n}\left(u_{nm}\right)$ at pixel positions $u_{nm}$ where $m=1,\ldots ,M_{n}$ with $M_{n}$ being the number of pixels in the *n*th image. Typically, the number of pixels $M_{n}$ is identical for all projection images.

A simple image model is to assume pixelwise identically and independently distributed Gaussian noise in the image formation (2), such that the likelihood of the *n*th image is

$$\text{Pr}\left(g_{n}|x,R_{n},t_{n},\gamma_{n},\alpha_{n},\tau_{n}\right)={\left(\frac{\tau_{n}}{2\pi }\right)}^{M_{n}/2}\text{exp}\left\{-\frac{\tau_{n}}{2}\overset{M_{n}}{\underset{m=1}{\sum }}{\left[g_{nm}-\alpha_{n}-\gamma_{n}\underset{k}{\sum }\phi_{2}\left(u_{nm};PR_{n}x_{k}+t_{n},\sigma^{2}\right)\right]}^{2}\right\}$$ (11)

where $\tau_{n}>0$ is the precision of the image, and $\alpha_{n},\gamma_{n}$ are an offset and a scaling factor (the constant weight $w_{k}=1/K$ has been absorbed by the scaling factor $\gamma_{n}$). The two-dimensional translation $t_{n}$ accounts for a shift of the image. These three to five nuisance parameters per image (depending on whether shifts $t_{n}$ are fitted or not) need to be estimated in addition to the particle positions $x=\left\{x_{k};k=1,\ldots ,K\right\}$ and the rotations $R=\left\{R_{n};n=1,\ldots ,N\right\}$. Model (11) is an idealized image formation model. It ignores important effects such as the CTF or correlated noise that are highly relevant for cryo-EM applications.

The second model also uses a kernel expansion of the input image motivated by the fact that ideally, according to our image model, the projection image should also be a mixture of spherical Gaussians (Eq. 10). In a preprocessing step, we fit a point cloud $Y_{n}=\left\{y_{nm}\in {\mathbb{R}}^{2};m=1,\ldots ,M_{n}\right\}$ to the *n*th input image $g_{n}$ such that

$$g_{n}\left(u\right)\approx \alpha_{n}+\gamma_{n}\overset{M_{n}}{\underset{m=1}{\sum }}\phi_{2}\left(u;y_{nm},\sigma_{n}^{2}\right)$$ (12)

Typically, we choose $M_{n}=M$ but this is not a requirement. Again, model (12) does not account for the CTF or other important effects in cryo-EM image formation. In each projection direction, the 2D point cloud can be blurred to a different degree captured by the width $\sigma_{n}$. The Supplementary Material details how projection images can be converted to point clouds; *Representation of projection images by point clouds* in Results shows a practical example for further illustration.

As in Joubert and Habeck (2015), we model the 2D point clouds as samples from the projected 3D volume:

$$\text{Pr}\left(Y_{n}|x,R_{n},t_{n},\sigma_{n}\right)=\overset{M_{n}}{\underset{m=1}{\prod }}\frac{1}{K}\overset{K}{\underset{k=1}{\sum }}\phi_{2}\left(y_{nm};PR_{n}x_{k}+t_{n},\sigma_{n}^{2}\right)$$ (13)

In the following, we will denote all nuisance parameters, i.e. all parameters except particle positions and rotations, collectively by ξ. In case of the image likelihood (11), we have $\xi =\left\{\left(\alpha_{n},\gamma_{n},\tau_{n},t_{n}\right);n=1,\ldots ,N\right\}$. In case of the point cloud likelihood (Eq. 13), we have $\xi =\left\{\left(\sigma_{n},t_{n}\right);n=1,\ldots ,N\right\}$. Moreover, we will denote both likelihoods as Pr(D|x,R,ξ) where *D* are the data (projection images or 2D point clouds).

#### 2.2.2 Priors

After incorporating our prior beliefs about the model parameters, we are able to derive the posterior distribution by invoking Bayes’ theorem:

$$\text{Pr}\left(x,R,\xi |D\right)=\frac{\text{Pr}\left(D|x,R,\xi \right)\;\text{Pr}\left(x,R,\xi \right)}{\text{Pr}\left(D\right)}$$ (14)

where Pr(x,R,ξ) is the prior which we assume to factor into

Pr(x,R,ξ)=Pr(x)Pr(R)Pr(ξ) (15)

The normalization factor Pr(D) is the model evidence, which can be ignored if we are only interested in parameter estimation.

We use standard priors for the nuisance parameters: Jeffreys priors for precisions $\tau_{n}$ and $1/\sigma_{n}^{2}$. The prior for the scaling factors and offsets are flat. Note that these priors are improper (i.e., not normalizable). Since we are only interested in parameter estimation, this does not pose a problem. The priors for the scaling factor and offset could be improved. For example, cryo-EM images are often normalized such that the mean intensity is zero and the standard deviation is one. It is possible to express this information as a prior on the offset and scaling factor. The Supplementary Material provides more details about these priors. For the image shifts $t_{n}$, a zero-centered two-dimensional Gaussian distribution is a reasonable choice.

Typically, biomolecules orient themselves randomly in the ice layer that is imaged by cryo-EM. Therefore, we choose a uniform distribution over SO(3):

$$\text{Pr}\left(R\right)=\overset{N}{\underset{n=1}{\prod }}\text{Pr}\left(R_{n}\right)\propto 1$$ (16)

These priors are proper, because the rotation group is compact.

In our previous work (Joubert and Habeck, 2015), we used a zero-centered Gaussian prior for all particle positions $x_{k}$ to ensure that prior and likelihood are conjugate, which enabled the derivation of closed-form updates for the component means. However, this prior is very unrealistic, if we think of the Gaussian basis functions as massive particles that should not occupy the same region in space (excluded volume), but rather repel each other. Since the packing of biomolecular structures is reminiscent of fluids (Liang and Dill, 2001), the prior should favor particle configurations that show similar packing characteristics. To model repulsive interactions between particles, we use a Boltzmann distribution over the positions $x_{k}$ involving a soft repulsive interaction potential E(x):

$$\text{Pr}\left(x_{1},\ldots ,x_{K}\right)\propto \text{exp}\left\{-\beta E\left(x_{1},\ldots ,x_{K}\right)\right\}$$ (17)

Furthermore, the particles are confined to a box with soft boundaries (Habeck, 2017). Pairs of particles repel each other if the distance is smaller than the particle diameter 2R where *R* is the effective particle radius. We choose a quartic repulsion which is commonly used in NMR structure calculation:

$$E\left(x_{1},\ldots ,x_{K}\right)=\underset{k<k^{\prime }}{\sum }\;\left[\;\left|\left|x_{k}-x_{k\prime }\right|\right|\leq 2R\right]{\left(1-\frac{\left\|x_{k}-x_{k}\prime \right\|}{2R}\right)}^{4}$$ (18)

where [⋅] is the Iverson bracket. Given the total number of atoms *L* of the system, the particle radius can be predicted for a desired number of particles *K* by using the relation

$$R\approx 0.92\;{\left(L/K\right)}^{0.42}\;\text{Å}.$$ (19)

Using a configurational temperature estimator (Mechelke and Habeck, 2013), the inverse temperature is estimated to β≈175. The estimates for *R* and *β* are based on an analysis of several biomolecular structures at different levels of coarse graining. See Supplementary Material for details.

Since the excluded-volume term (Eq. 18) is purely repulsive, we add a radius of gyration term such that the overall prior for particle positions is

$$\text{Pr}\left(x_{1},\ldots ,x_{K}\right)\propto \text{exp}\left\{-\beta E\left(x_{1},\ldots ,x_{K}\right)\right\}\;\text{exp}\left\{-\alpha R_{g}\left(x\right)\right\}$$ (20)

where $R_{g}\left(x\right)$ is the radius of gyration of the coarse-grained structure x and *α* a positive constant. The radius of gyration term imposes a weak preference for compact structures and prevents configurations with isolated particles that do not contact another particle. In our experiments, we set α=10 Å; in principle, we could estimate *α* by using techniques similar to those used in the estimation of *β*. But since *α* does not have a strong impact on the final structure, we restricted ourselves to a single fixed value for *α*.

### 2.3 Inference

Bayesian random tomography employs MCMC sampling from the posterior distribution (14). We use a Gibbs sampling strategy (Geman and Geman, 1984) where each group of parameters, the particle positions x, the rotations R and the nuisance parameters *ξ*, is updated separately while clamping the other parameters to their current values. To update the nuisance parameters, we use standard samplers for generating Gamma variates and normally distributed random variables (more details can be found in the Supplementary Material). However, the conditional posteriors of the particle positions x and the rotations R are not of a standard form and need to be updated with more sophisticated algorithms.

#### 2.3.1 Sampling Particle Positions With Hamiltonian Monte Carlo

To sample the particle positions, we use Hamiltonian Monte Carlo (HMC) (Neal, 2011). The conditional posterior distribution over particle positions is

Pr(x|R,ξ,D)∝Pr(D|x,R,ξ) Pr(x)

In HMC, −logPr(x|R,ξ,D) defines a potential energy over configuration space that is composed of an attractive term −logPr(D|x,R,ξ) matching particle positions to the projection data, and a repulsive contribution −logPr(x) stemming from the excluded-volume term (18). For fixed rotations and nuisance parameters, the particle positions undergo Hamiltonian dynamics following the gradient of −Pr(x|R,ξ,D) during a short leapfrog integration. The resulting configuration is accepted or rejected according to the Metropolis criterion.

#### 2.3.2 Sampling Rotational Parameters With Metropolis-Hastings

A challenging problem is to estimate the rotations. Because the projection images are statistically independent of each other, the problem decomposes into *N* subproblems:

$$\text{Pr}\left(R_{n}|x,\xi ,D\right)\propto \text{exp}\left\{-\frac{\tau_{n}}{2}\overset{M_{n}}{\underset{m=1}{\sum }}{\left[g_{nm}-\alpha_{n}-\gamma_{n}\overset{K}{\underset{k=1}{\sum }}\phi_{2}\left(u_{nm};PR_{n}x_{k}+t_{n},\sigma^{2}\right)\right]}^{2}\right\}$$ (21)

if projection images $g_{n}$ are fitted directly, or

$$\text{Pr}\left(R_{n}|x,\xi ,D\right)\propto \overset{M_{n}}{\underset{m=1}{\prod }}\overset{K}{\underset{k=1}{\sum }}\phi_{2}\left(y_{nm};PR_{n}x_{k}+t_{n},\sigma_{n}^{2}\right)$$ (22)

if we fit 2D point clouds. In Joubert and Habeck (2015), we introduced assignment variables such that the conditional posterior (22) is replaced by the matrix von Mises-Fisher distribution, which can be simulated in a straightforward fashion (Habeck, 2009). However, because the assignment variables are highly coupled to the other parameters, this strategy converges only slowly to the next local minimum. Moreover, there is no flexibility regarding the likelihood function.

We use the Metropolis-Hastings (MH) algorithm (Liu, 2001) to estimate the rotation matrices. We parameterize rotation matrices using unit quaternions (Horn, 1987) and propose new quaternions by adding a random perturbation that is sampled from a uniform distribution. We run 10 MH steps to update the quaternions representing each projection direction in every Gibbs sampling iteration and adapt the step-size automatically: Upon acceptance, the step-size increases by multiplying it with a factor of 1.02; in case of rejection, the step-sizes decreases by a factor of 0.98. This rule results in an acceptance rate of approximately 50%. We use this sampling algorithm to simulate both types of conditional posteriors (21) and (22).

### 2.3.3 Global Sampling of Rotational Parameters

Since the MH algorithm achieves only local sampling of probability distributions, we occasionally scan all rotations systematically. The unit quaternions are elements of the 3-sphere, the unit sphere embedded in the four-dimensional space. To evenly cover rotation space, we discretize the 3-sphere using the 600-cell (Coxeter, 1973). The 600-cell is composed of even sized tetrahedra whose corners lie on the unit sphere. By projecting the center of a tetrahedron onto the unit sphere we obtain a unit quaternion parameterizing a valid rotation matrix. Due to the degeneracy of the quaternions we only have to consider the upper half of the 4D sphere that is covered by 330 tetrahedra at the coarsest level of discretization. To obtain a finer tessellation of SO(3), we can split each tetrahedron into eight tetrahedra whose corners again lie on the 4D unit sphere. By default, we use a frequency of 0.1 to run a global rotation scan. The conditional posterior is evaluated for all rotations and then sampled from the discrete distribution.

The source code and scripts for reproducing the tests are available at github.com/michaelhabeck/bayesian-random-tomography.

## 3 Results

### 3.1 Sampling Tests

To test MCMC strategies for inferring particle positions and rotations, we use the structure of the GroEL/GroES complex. This system has been studied extensively with cryo-EM. Since our focus is mainly on algorithmic aspects, we first use simulated data that exactly follow our probabilistic model. To generate input point clouds in 2D, we use the crystal structure of GroEL/GroES (PDB code 1aon; 58,674 atom coordinates in total). The 2D point clouds are generated by projecting the 3D positions of every 10th Carbon-alpha atom (802 points in total) along 35 random directions into 2D. We also generated corresponding projection images by blurring the point clouds with a Gaussian filter of width 5 Å.

#### 3.1.1 Sampling Particle Positions and Precisions With Fixed Rotations

We first studied the performance of sampling particle positions by fixing the rotations to the correct values and sampling only the particle positions and the precisions of the projection data. HMC sampling of particle positions started from a random initial configuration for *K* ranging between 50 and 1,000 particles. In all of our HMC experiments, the number of leapfrog steps was set to 10, whereas the step-size was adjusted automatically. The precisions $1/\sigma_{n}^{2}$ follow Gamma distributions and can be sampled directly.

**FIGURE 2:**
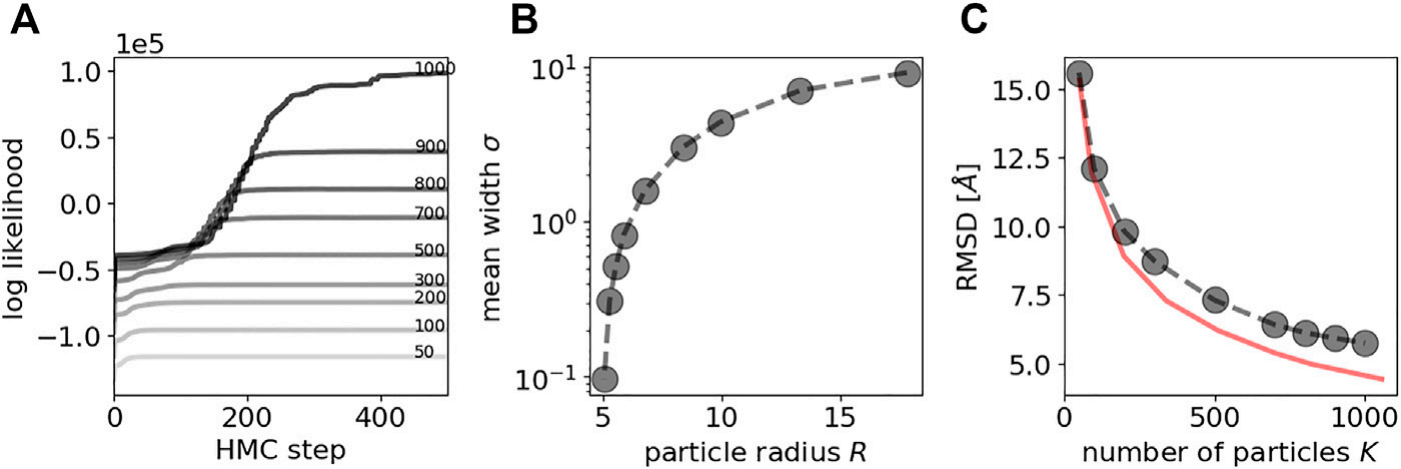
HMC sampling of particle positions with fixed rotations for a simulated data set of GroEL/ES. A Evolution of the log likelihood during HMC sampling. The larger the number of particles *K*, the higher is the final log likelihood. Increasing darkness indicates larger number of particles. Line annotations also indicate the number of particles. B Average standard deviation (computed over all 35 input point clouds) vs. the size of the particle *R*. C RMSD between Carbon-alpha positions of the crystal structure and the coarse-grained models inferred with HMC. As a reference, the RMSD between the Carbon-alpha positions and the coarse-grained versions of the crystal structures is shown as red curve.

Figure 2A shows the evolution of the log likelihood achieved by the particle system during HMC. After roughly 200 to 500 HMC steps (depending on *K*), the particle cloud reproduces the input data well, which is reflected in high values of the log likelihood. The sampled particle configurations are very similar to the true structure at the same level of coarse graining. Successful sampling of Pr(x|R,ξ,D) with HMC is observed reliably for many different initial particle configurations.

It is clear that an increasing number of particles *K* results in a higher goodness of fit, which is obvious from Figures 2A,B showing the average standard deviation $\sigma_{n}$ of the point cloud likelihood (Eq. 13) as a function of particle radius: A higher number of particles *K* results in more flexible models that result in a better goodness of fit and higher precision. These findings indicate that HMC is highly suited to sample particle configurations.

Figure 2C shows the accuracy of the coarse-grained models inferred from the projection data with HMC. The accuracy is quantified by the root mean square deviation (RMSD) between corresponding positions in a reference structure and a coarse-grained model. Here, our reference structure is the atomic structure of GroEL/ES reduced to the positions of 8,015 Carbon-alpha atoms listed in the PDB entry 1aon. To compare this structure with a coarse-grained model, positions in the atomic structure are assigned to positions in the coarse-grained model that are closest in 3D space. There are two factors that contribute to this measure of accuracy: the level of coarse graining as well as the performance of posterior sampling based on the 2D projection data. To disentangle both contributions, we also show the accuracy between the crystal structure and its coarse-grained versions (obtained with the DP-means algorithm by Kulis and Jordan (2012); also see the Supplementary Material). This curve shows that coarse-grained models of GroEL/ES using 1,000 particles achieve an accuracy of about 4.6 Å, whereas an ultra coarse-grained model based on only 50 particles is on average 15.5 Å away from any Carbon-alpha atom in the crystal structure. For very high levels of coarse graining (small *K*), the models inferred with HMC reach the maximum accuracy that is possible at this level of coarse graining. With increasing number of particles *K*, the gap in accuracy widens but is still similar to the maximum attainable value. For example, with K=1000 the model obtained with HMC achieves an RMSD of 5.7 Å, whereas the coarse grained model obtained directly from the crystal structure achieves an accuracy of 4.6 Å.

If we estimate particle configurations from projection images instead of point clouds, we obtain similar results. Supplementary Figure S4 shows the log likelihood and cross-correlation coefficients obtained with different numbers of particles, again ranging between 50 and 1,000. The evolution of the log likelihood indicates that the HMC sampler seems to converge even faster compared to a simulation based on point cloud data: within 20–150 HMC steps the log likelihood plateaus. The accuracy of the structure after 500 HMC steps is similar to or better than the accuracy of the particle models fitted against 2D point clouds and almost reaches the accuracy of the coarse-grained models derived from the crystal structure. Supplementary Figure S5 shows FSC curves for all 3D models. For the same number of particles, the FSC curves are similar with a slight preference for the image-based models when using larger numbers of particles. The resolution ranges from 12.2 Å (50 particles) to 4.5 Å (1,000 particles). Supplementary Table S1 shows resolution estimates for all models.

#### 3.1.2 Sampling Rotational Parameters and Precisions With Fixed Particle Positions

To test our rotational sampling approach, we fixed the particle positions to an ultra coarse-grained structure (K=200) of GroEL/ES. Although each rotation can be updated independently of the other rotations, and each conditional posterior (given either by Eqs. 21 or 22) is only a four-dimensional probability distribution over the quaternions, the sampling problem is still challenging due to its multimodality. Since Metropolis-Hastings (MH) is a local sampling algorithm, it tends to become trapped in subordinate modes of the conditional posterior, which are typical for rigid registration problems. As a result, running MH on the conditional posteriors is not sufficient to reliably recover the rotation matrices.

**FIGURE 3:**
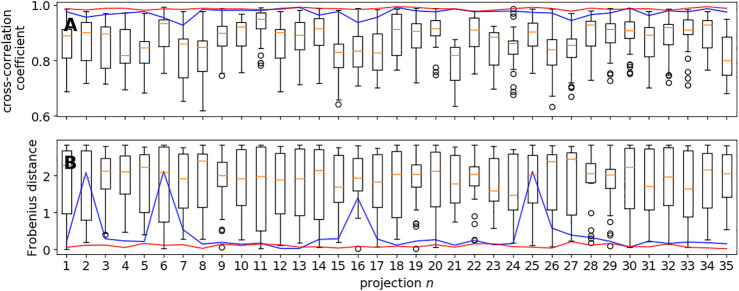
Global vs. local sampling of orientational parameters. Shown are the cross-correlation coefficients **(panel A)** and Frobenius distances **(panel B)** for each of the 35 input directions achieved with local sampling based on the MH algorithm and global sampling using a regular discretization of the 3-hemisphere. The blue curve shows the results obtained with the coarsest covering based on 330 unit quaternions; the red curve shows the results obtained with a finer covering (2,460 quaternions). The box plots show the variability within 30 trials of MH starting from random rotations.

Figure 3A shows the cross-correlation coefficients for the 35 projection images obtained with global rotational sampling in comparison with MH runs starting from 30 random rotations. Global rotational sampling was based on the first two discretizations of the 3-hemisphere using 330 and 2,640 quaternions, respectively. The number of local sampling attempts was set to 30 so as to match the speed of global sampling at the finer level. That is, the coarse sampling based on 330 quaternions is approximately 8 times faster than the 30 local sampling trials. As evidenced by Figure 3A, global sampling is capable of finding rotation matrices that yield high cross-correlation coefficients, whereas MH alone fails to do so in a systematic fashion. Figure 3B shows the Frobenius distances (ranging from 0 to a maximum of $2\sqrt{2}$) between the true rotation matrix and the estimated rotation matrices. Again, global rotational sampling achieves more accurate rotations, whereas the distances scatter largely for the local MH trials. These findings suggest that global rotational sampling is indispensable for Bayesian random tomography in agreement with our previous findings (Joubert and Habeck, 2015) where we had to resort to repeated Gibbs sampling runs.

Before we study sampling of the full posterior distribution (all parameters R, x and ξ are unknown), we will first outline how experimental projection images can be converted to 2D point clouds that are suitable for our approach to random tomography.

### 3.2 Representation of Projection Images by Point Clouds

Experimental projection data are typically presented as projection images rather than point clouds. In this subsection, we discuss how to convert 2D projection images to 2D point clouds that are suitable for our Bayesian random tomography approach. We discuss this for a cryo-EM data set, but similar techniques are also applicable to other data, as we will demonstrate later.

The projection properties of mixtures of spherical Gaussians (Eq. 10) suggest to also represent the projection image as a mixture of Gaussians. Our model can only capture nonnegative intensities. Therefore, we first have to choose a suitable threshold θ above which image intensities are considered real signal. The threshold will be used to construct a binary mask: the intensities of pixels that are part of the mask will be shifted by θ such that their shifted intensities are nonnegative; the intensities of pixels that are not part of the mask will be set to zero (i.e., they will be ignored in the construction of the point cloud). A simple choice of θ for class averages from cryo-EM is the median intensity, but a different choice might be more suitable for other types of images.

**FIGURE 4:**
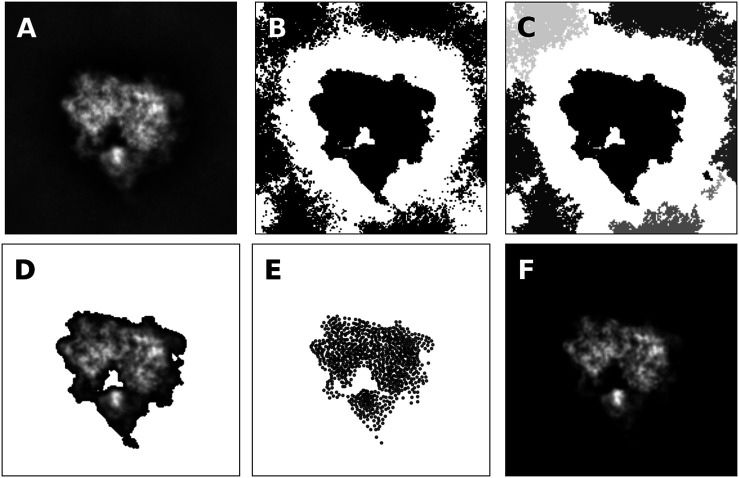
Representation of projection images by 2D point clouds **(A)** Class average of the 80S ribosome **(B)** Mask obtained by thresholding image intensities greater than the median intensity. Black pixels are part of the mask **(C)** Clustering of pixels that are part of the mask. Pixels that form a connected component are grouped together and shown in different grayscale colors **(D)** Pixels that form the most central connected components with shifted image intensities **(E)** 2D point cloud composed of 1,000 particles obtained by running the Expectation Maximization algorithm **(F)** Model image according to Eq. 10. The cross-correlation coefficient between the model and the original image is 95.8%. If only pixels are considered that are part of the mask indicating the central connected component, the cross-correlation coefficient increases to 99.6%.

An example of the thresholding procedure is shown in Figure 4B for a class average showing the projection of the 80S ribosome (shown in Figure 4A). Black pixels indicate pixels with intensity above the median. By looking at the mask, it is clear that only the central pixels forming a connected component carry signal.

Next, we identify pixels that form connected components. Again this applies to cryo-EM images; other types of images might require a different treatment to construct a suitable mask. To identify signal pixels that form a connected component, we convert the thresholded image to an undirected graph G=(V,ℰ) where the pixels with intensities above the threshold are the vertices $V=\left\{u_{m};g\left(u_{m}\right)>\theta ,m=1,\ldots ,M\right\}$. Edges are introduced between all pairs of pixels that are nearest neighbors on the 2D square lattice, i.e. their Euclidean distance is smaller than or equal to one pixel:

$$ℰ=\{\left(i,j\right)\in {\left\{1,\ldots ,|\nu |\right\}}^{2};∥u_{i}-u_{j}∥\leq 1\}.$$

As shown in Figure 4C, multiple connected components are typically found in the masked pixels. Since cryo-EM class averages are often centered, we pick the connected component whose center of mass is closest to the image center. The selected pixels including their intensity (shifted by θ) are shown in Figure 4D.

To obtain a particle-based representation of the central connected component, we run the Expectation Maximization algorithm (details in Supplementary Material). Figure 4E shows the estimated point cloud using 1,000 particles. The estimated standard deviation of the Gaussian is 1.34 pixels. The density generated by the 2D particles is shown in Figure 4 and correlates highly with the original image and the masked image. Supplementary Figure S1 shows more examples of class averages represented as 2D point clouds.

### 3.3 3D Reconstruction by Sampling the Full Posterior Distribution

We applied Bayesian random tomography to three real datasets, two cryo-EM datasets and one dataset from stochastic microscopy experiments visualizing marine microorganisms. In these applications, we sampled the joint posterior distribution of all unknown parameters, particle positions $x_{k}$, rotations $R_{n}$ and nuisance parameters ξ, with the MCMC techniques discussed above. We started our reconstruction simulations from spherical random structures and random rotations and did not observe any dependence on the initial values.

The first dataset is comprised of 400 2D class averages of the 80S ribosome computed with SIMPLE2 (Elmlund and Elmlund, 2012) from cryo-EM micrographs (EMPIAR-10028); the size of the images is 80×80 pixels, the pixel size is 2.68 Å. The class averages are part of a SIMPLE2 tutorial and publicly available at https://simplecryoem.com/SIMPLE3.0/old_pages/2.5/data/simple2.5tutorials.tgz. Figure 4 and Supplementary Figure S1 show some example images and the 2D point clouds that were generated with the procedure outlined in subsection 3.2. Class averages were converted to 2D point clouds each composed of 1,000 points. Because the dataset is highly redundant, we only used the first 50 class averages and point clouds in the posterior simulations.

**FIGURE 5:**
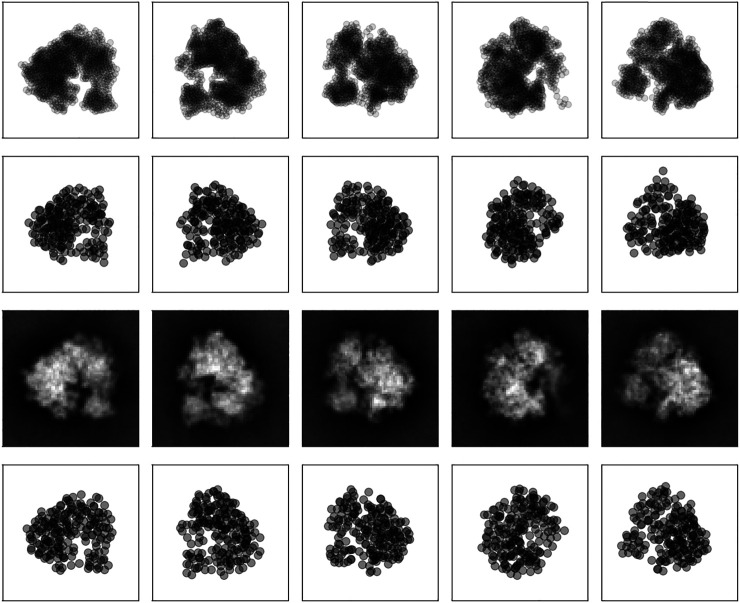
2D projections of the 80S ribosome. First row: point clouds derived from class averages. Each projection image is represented by 1,000 points. Second row: 2D projections of the coarse-grained model calculated with Bayesian random tomography based on 2D point clouds. Third row: Class averages. Bottom row: 2D projections of the coarse-grained model calculated with Bayesian random tomography based on class averages.

We used K=200 and K=1000 particles with a radius of R=16.4 and R=8.4 Å, respectively to fit the ribosome point clouds. We ran 500 iterations of Gibbs sampling with the global strategy for the rotational parameters and HMC for the particle positions. Figure 5 shows five input point clouds and the projected model after convergence. We observe a good agreement between the experimental point clouds and the model point clouds with an RMSD ranging between 6.4 Å and 9.8 Å and an average of 7.7±0.7 Å.

**FIGURE 6:**
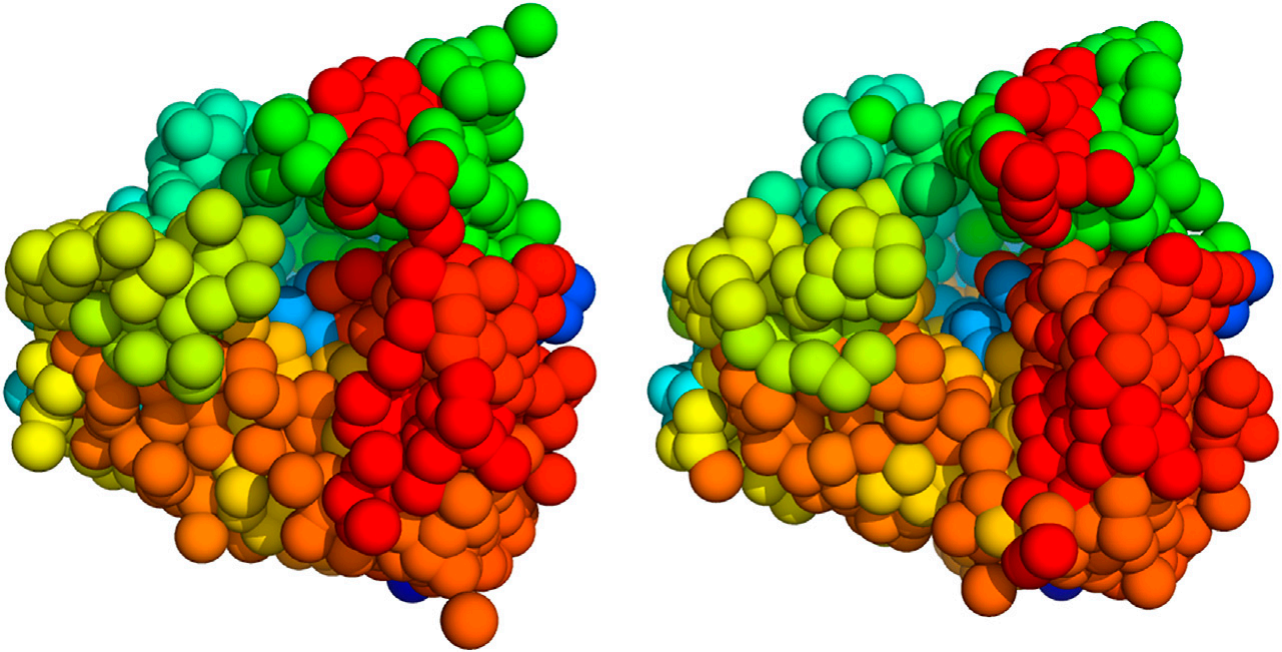
3D models of the 80S ribosome **(Left)** 1,000 particle model inferred with Bayesian random tomography **(Right)** Initial model computed with PRIME. The particles are sorted such that spatially close particles have similar indices. By using Pymol’s chainbow command, we can then visualize the particle models such that substructures are better visible.

We also compared our 3D coarse-grained model of the 80S ribosome with a structure obtained with PRIME (Elmlund et al., 2008). To simplify the comparison, we converted the density map obtained with PRIME to a structure made up of 1,000 particles. The indices of the particle models were ordered such that spatially close particles have similar particle indices (which can be achieved, for example, by solving a traveling salesman problem using the matrix of inter-particle distances as input). Both structures show similar features (Figure 6); an FSC analysis reveals a resolution of 15.5 Å using the 0.143 criterion (Supplementary Figure S6).

We also ran simulations based on the first 50 class averages rather than 2D point clouds using 200 up to 12,000 particles. Again, we ran 500 steps of Gibbs sampling where the rotational parameters were updated globally with a frequency of 0.1. Projections of the 200 particle model are shown in the bottom rows of Figure 5. The cross-correlation coefficient between the class averages and the model images ranges between a minimum and maximum value of 90%–96% with an average of 94±1%. For comparison, we also report the RMSDs to the particle clouds which range between 6.1 Å and 13.1 Å and an average of 8.3±3.0 Å.

**FIGURE 7:**
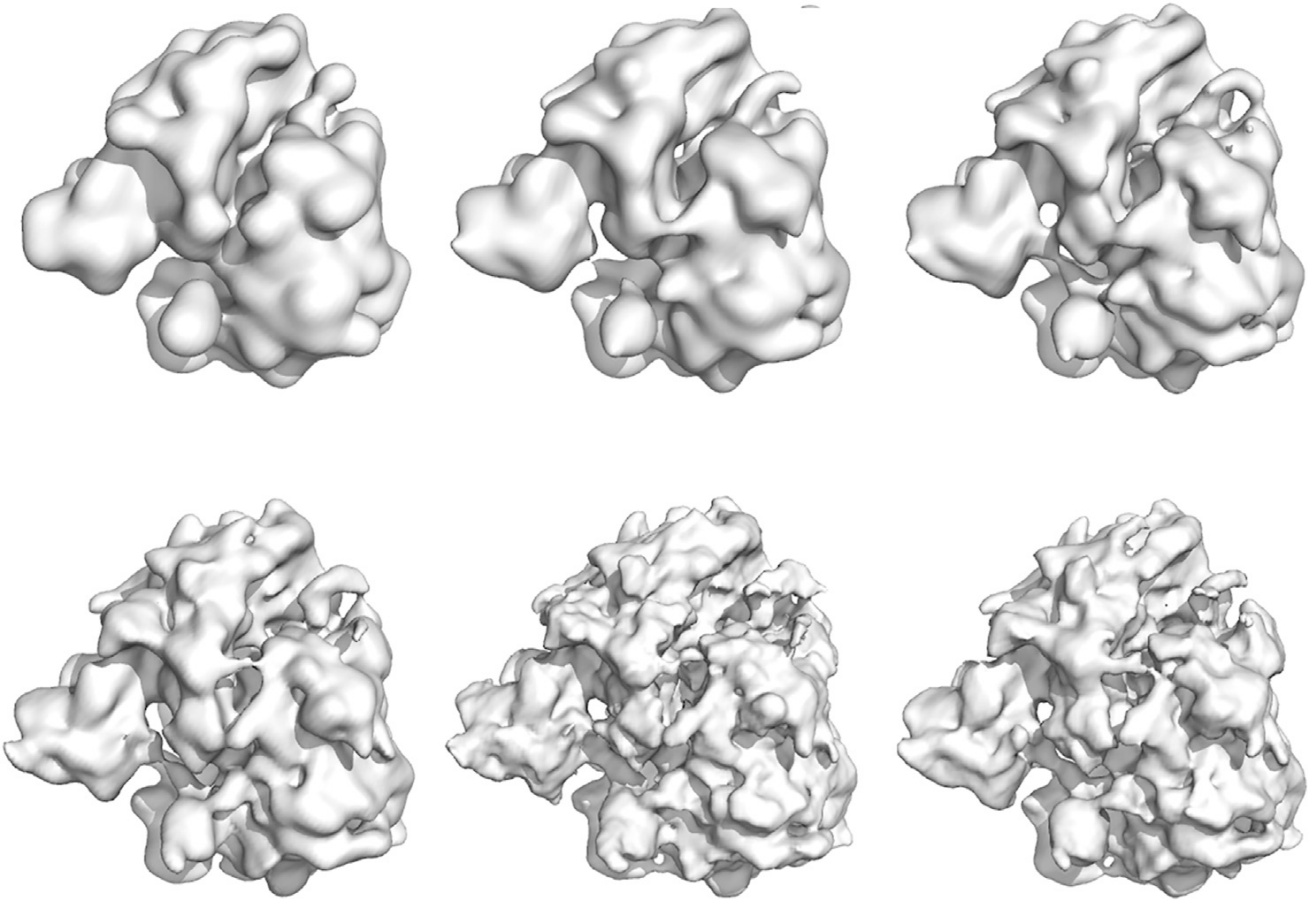
Density maps of the 80S ribosome obtained with Bayesian random tomography using 50 class averages as input. Top row: 200, 1,000, 2000 particles **(left to right)**. Bottom row: 4,000, 8,000, 12,000 particles **(left to right)**.

Using the last 100 particle configurations, we also generated density maps for each simulation and compared them to the high-resolution reconstruction EMD-2660 (Wong et al., 2014). The density maps are shown in Figure 7. To assess the quality of the particle models, we computed the FSC between the high-resolution map and the model maps (Supplementary Figure S6). Based on the 0.143 criterion, the resolution of the particle models ranges from 23.6 Å (200 particles) to 10.6 Å (12,000 particles). For comparison, the reconstruction obtained with SIMPLE reaches a resolution of 6.2 Å based on 200 class averages. More details about the quality of the reconstruction and computation times can be found in the Supplementary Material (Supplementary Tables S2, S3).

The posterior samples can be also used to assess the uncertainty of the particle models in the form of structural error bars. To carry out uncertainty quantification, the particle models first need to be superimposed and a correspondence between particles across different samples has to be established. We solve these two tasks by using the Iterative Closed Point (ICP) method followed by a linear assignment step where particle distances between superimpose clouds are used as a cost. Supplementary Figure S7 shows an example for structures based on 200 and 2000 particles. The distribution of uncertainties is inhomogeneous. Highly uncertain particles tend localize on the surface of the 200-particle model. The 2000-particle model shows smaller variations in the uncertainty of particle positions. So the large variations in the uncertainties of the 200-particle model might also be caused by the small number of particles.

The second cryo-EM dataset comprises 16 class averages of beta-galactosidase. These images are part of a RELION tutorial and available at ftp://ftp.mrc-lmb.cam.ac.uk/pub/scheres/relion31_tutorial_precalculated_results.tar.gz. The class average based on the data from EMPIAR-10204. The size of the images is 60×60 pixels, the pixel size is 3.54 Å. In this test, we inferred the structure from the images directly using likelihood (11) without converting the class averages to 2D point clouds.

Similar to the ribosome simulations we used 500 steps of Gibbs sampling with occasional global sampling of the rotational parameters to infer the coarse-grained structure of beta-galactosidase. We inferred structural models for systems with 100 up to 2000 particles.

**FIGURE 8:**
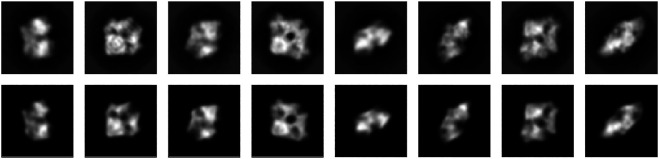
2D projections of beta-galactosidase. **Top row:** eight (out of 16) projection images (RELION class averages). **Bottom row:** Projection images calculated with Bayesian random tomography using 500 particles.

The top row of Figure 8 shows the first eight class averages that were used as an input for particle-based random tomography. The bottom row shows the projection images of a model composed of 500 particles that was obtained with sampling the full posterior distribution. Starting from a random initial structure and rotations, our sampling algorithm estimates a model structure and orientations that reproduce the experimental images closely with cross-correlation coefficients ranging between 94.7% and 97.5% and an average of 95.9±0.01%.

**FIGURE 9:**
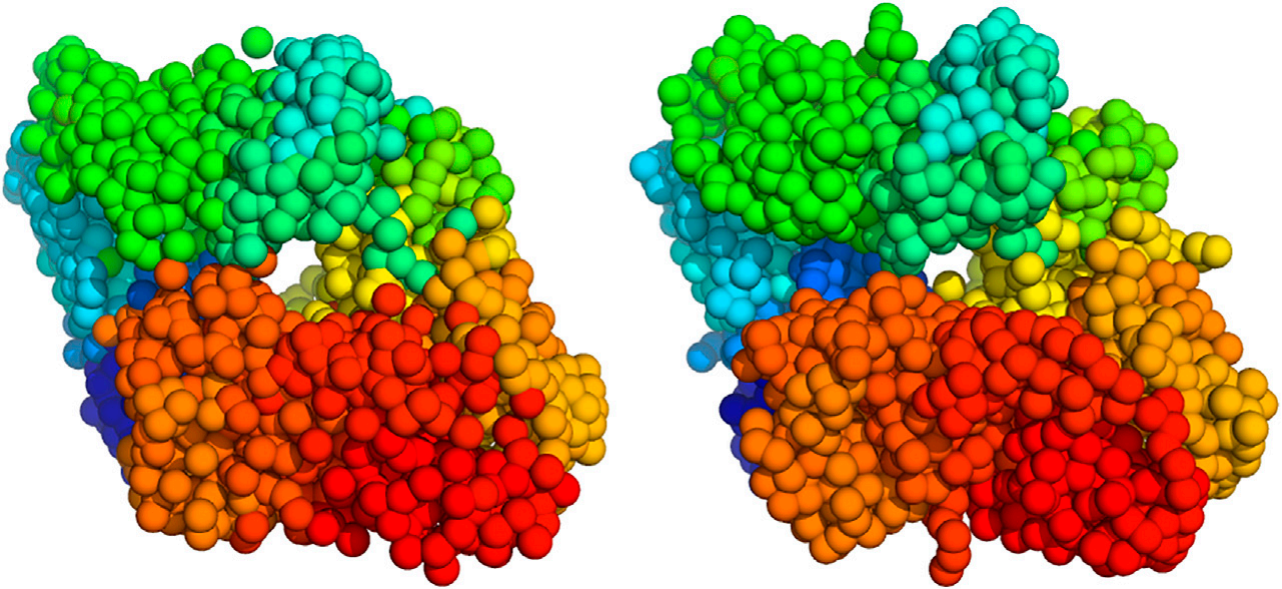
3D models of beta-galactosidase **(Left)** 2000 particle model inferred with Bayesian random tomography **(Right)** Coarse-grained model of the atomic structure (PDB code 1jz8).

We compared the structure inferred with Bayesian random tomography against a high-resolution crystal structure (PDB code 1jz8) and a near-atomic cryo-EM reconstruction (EMD-5995). To enable this comparison, we converted the PDB structure to a 3D point cloud composed of 2000 particles. Correspondences between particles in our model and the model based on the crystal structure were established as in the calculation of the RMSD. Figure 9 shows both models. The RMSD between our particle model and the Carbon-alpha atoms of the high-resolution structure 1jz8 is 3.4 Å. For comparison, we also report the RMSD between 1jz8 and its coarse-grained version (shown on the right of Figure 9) which is 2.4 Å. Bayesian random tomography achieves a similar accuracy by inferring a 3D model from the class averages as direct coarse graining of the high-resolution structure. Supplementary Figure S8 shows density maps for all of the five simulations. By comparison with the high-resolution reconstruction (EMD-5995) we assess the resolution of the models to range between 25 Å (100 particles) and 11.5 Å (2000 particles). For comparison, the initial model from RELION achieves a resolution of 9.8 Å (Supplementary Figure S9 shows the corresponding FSC curves).

To assess the impact of the Boltzmann prior (Eq. 17), we ran two posterior simulations using 200 and 1,000 particles with the inverse temperature set to zero (i.e. the repulsive inter-particle energy is switched off). The quality of the reconstructed density map is largely unaffected by this change. For the 200 particles model, the average cross-correlation with Boltzmann prior is 94.7±1.1%; without the Boltzmann prior we have 95.7±0.9%. For the 1,000 particles model, these averages are 95.5±1.5% (with Boltzmann prior) and 95.9±1.5% (without Boltzmann prior). A comparison of the FSC curves obtained with and without Boltzmann prior confirms this finding (Supplementary Figure S11). The estimated resolution of the 200-particle model is 20.5 (19.4) Å with (without) Boltzmann prior; the 1000-particle model achieves a resolution of 12.0 (11.6) Å with (without) Boltzmann prior.

However, the Boltzmann prior has a strong effect on the packing of particles as assessed by the radial distribution functions (Supplementary Figure S11). With Boltzmann prior, the radial distribution shows a prominent peak close to the particle diameter, which is indicative of local order similar to a fluid. Without the Boltzmann prior, this peak disappears and we observe an enrichment of very short distances indicating a physically unrealistic particle packing. If our goal is to reconstruct a single 3D density from a homogeneous dataset, introducing the Boltzmann prior is not harmful, but dispensable. Turning the argument around, we find that the Boltzmann prior is compatible with the data and does not result in a severe loss of fitting quality. We expect that the prior will become essential in more advanced 3D reconstruction tasks, in particular when facing conformational heterogeneity.

Finally, we applied our random tomography approach to a dataset that shows structures on length scales that are much larger than the length scales imaged in cryo-EM. Following the work by Levis et al. (2018), we downloaded *in situ* microscopy images of the marine plankton species Pyramimonas Longicauda; the data are available at https://darchive.mblwhoilibrary.org/handle/1912/7341. These mesoscopic organisms are transparent and therefore allow for 3D reconstruction from 2D microscopic images. Since the organism seems to be quasi symmetric, we selected out of the 121 projection images recorded in 2013, 16 representative images. The selected images cover most of the views that are present in the dataset.

**FIGURE 10:**
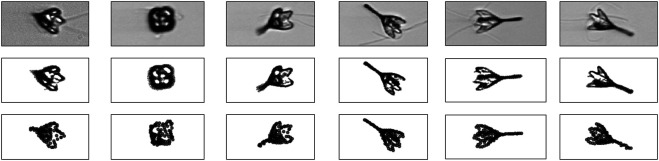
Stochastic microscopy images of a plankton species. **Top row:** six (out of 16) projection images. **Middle row:** 2D point clouds representing the image data. **Bottom row:** 2D projections of the particle model calculated with Bayesian random tomography.

The intensity of microscopic images $g_{n}$ is proportional to the transmissivity, which is related to the optical density of the object via an exponential transform. Therefore, to convert the images to 2D point clouds, we use the expectation maximization approach (see Supplementary Material) with weights proportional to $-\text{log}g_{n}>0$, since $g_{n}\in \left(0,1\right)$. The six out of the 16 selected images and their point cloud representations are shown in Figure 10. Each microscopic image was converted to 2D cloud composed of 1,000 points.

The fact that the magnification can vary from image to image requires that we extend the likelihood for 2D point clouds (13) (also Supplementary Equations S1, S2 in the Supplementary Material). These variations are accounted for by an additional factor that scales the coordinates of the projected model so as to match the 2D point cloud derived from the microscopic image. Moreover, we need to account for shifts in the image plane. These extensions increase the number of unknown parameters per image from four to eight: four quaternions parameterizing the unknown orientation, two translation parameters accounting for a shift, a scaling factor compensating variations in the magnification and a precision.

**FIGURE 11:**
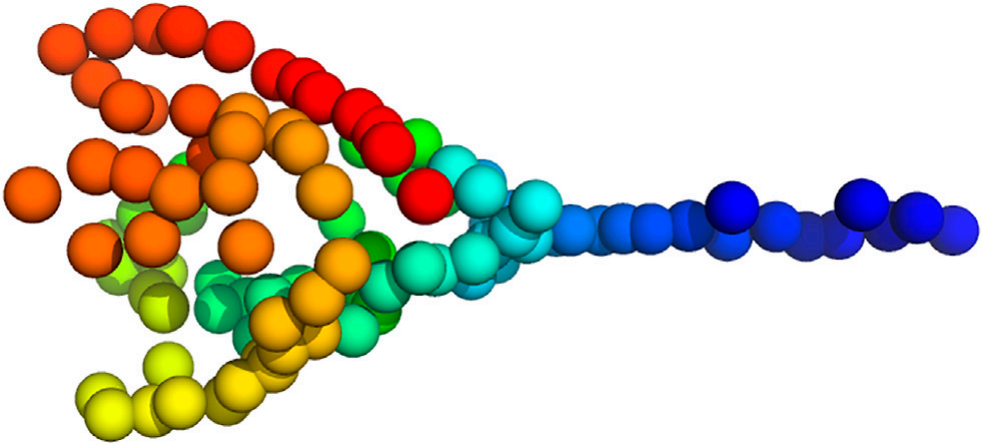
3D model of Pyramimonas Longicauda using 100 particles inferred from the point clouds shown in Figure 10.

Inference of a 3D particle model proceeded as before. We estimated a model composed of 100 particles from the 16 2D point clouds starting from a random structure and random rotations (the initial values for the scaling factors and translations were one and zero, respectively). Figure 11 shows a 3D model of the plankton species inferred with Bayesian random tomography.

## 4 Discussion

We outlined a Bayesian approach to random tomography, the problem of reconstructing a 3D structure from 2D views along unknown random directions. At the core of our approach is a representation of 3D volumes using a radial basis function kernel whose centers are our main inference parameters. We interpret the kernel centers as particle positions and use an excluded-volume prior to ensure that estimated particle configurations show a physically plausible packing. We demonstrated that coarse-grained models can be inferred from projection data (images or point clouds) with MCMC algorithms such as HMC and global sampling of the rotations.

In cryo-EM applications, our approach can be used to generate an initial model that can be refined further. So far, we tested the method only an class averages that displayed a high SNR. In future applications, we plan to explore the use of Bayesian random tomography from raw cryo-EM images and include the effect of the CTF into our model. Another route for extending the approach is account for conformational heterogeneity, which is one of the major bottlenecks in cryo-EM data processing. An interesting approach to characterize conformational variability in the presence of continuous flexibility has been proposed recently by Chen and Ludtke (2021) who use an autoencoder network with a Gaussian mixture model to represent conformational changes in a low dimensional latent space.

In all applications discussed in this paper, the number of particles K was fixed. An interesting question for future research is to estimate the number of particles based on the projection data. This might also provide a new way of measuring the resolution of the input data.

## Data Availability

Publicly available datasets were analyzed in this study. This data can be found here: https://darchive.mblwhoilibrary.org/handle/1912/7341 ftp://ftp.mrc-lmb.cam.ac.uk/pub/scheres/relion31_tutorial_precalculated_results.tar.gz https://simplecryoem.com/SIMPLE3.0/old_pages/2.5/data/simple2.5tutorials.tgz.
