# Supplementary material for: Bayesian Random Tomography of Particle Systems
Source: Front Mol Biosci. 2021 May 21;8:658269. doi: 10.3389/fmolb.2021.658269 (PMC8177743; doi:10.3389/fmolb.2021.658269)
Supplement: Supplementary file 1 [file DataSheet1.pdf]

# Supplementary Material

## 1 PROBABILISTIC MODEL AND INFERENCE ALGORITHMS

The likelihoods for both types of input data are detailed in the main text of the manuscript (Eqs. 11 and 13). In case we have to account for an additional image-wise magnification factor $s_n$ such as in the *in situ* microscopy application, these likelihoods generalize to

$$\Pr(g_n|\boldsymbol{x}, \boldsymbol{R}_n, \xi) = \left(\frac{\tau_n}{2\pi}\right)^{M_n/2} \exp\left\{-\frac{\tau_n}{2}\sum_{m=1}^{M_n}\left[g_{nm} - \alpha_n - \gamma_n \sum_k \phi_2\big(\boldsymbol{u}_{nm}; s_n(\boldsymbol{P}\boldsymbol{R}_n\boldsymbol{x}_k + \boldsymbol{t}_n), \sigma^2\big)\right]^2\right\} \tag{S1}$$

for images where $\xi_n = (\boldsymbol{t}_n, s_n, \gamma_n, \alpha_n, \tau_n)$ and

$$\Pr(Y_n|\boldsymbol{x}, \boldsymbol{R}_n, \xi_n) = \prod_{m=1}^{M_n} \frac{1}{K}\sum_{k=1}^{K} \phi_2\big(\boldsymbol{y}_{nm}; s_n(\boldsymbol{P}\boldsymbol{R}_n\boldsymbol{x}_k + \boldsymbol{t}_n), {\sigma_n}^2\big)\,. \tag{S2}$$

for 2D point clouds where $\xi_n = (\boldsymbol{t}_n, s_n, \sigma_n)$.

The Jeffreys' priors for the precision parameters are

$$\tau_n \sim 1/\tau_n$$

where in case of the point cloud likelihood (Eq. S2) $\tau_n = 1/\sigma_n^2$. The conditional posteriors of these parameters are Gamma distributions:

$$\tau_n \sim \tau_n^{M_n/2-1} \exp\left\{-\tau_n\chi_n^2/2\right\} \tag{S3}$$

where

$$\chi_n^2 = \sum_{m=1}^{M_n}\left[g_{nm} - \alpha_n - \gamma_n \sum_k \phi_2\big(\boldsymbol{u}_{nm}; s_n(\boldsymbol{P}\boldsymbol{R}_n\boldsymbol{x}_k + \boldsymbol{t}_n), \sigma^2\big)\right]^2$$

Efficient random number generators for Gamma-variates are offered by libraries such as `numpy.random` from the Python array processing library NumPy. The conditional posterior of the offset and scale is a two-dimensional Gaussian; to update these parameters, we generate a sample from the bivariate Normal distribution by calling `numpy.random.multivariate_normal`. The shifts $\boldsymbol{t}_n$ and magnification factors $s_n$ can be sampled with the Metropolis-Hastings algorithm.

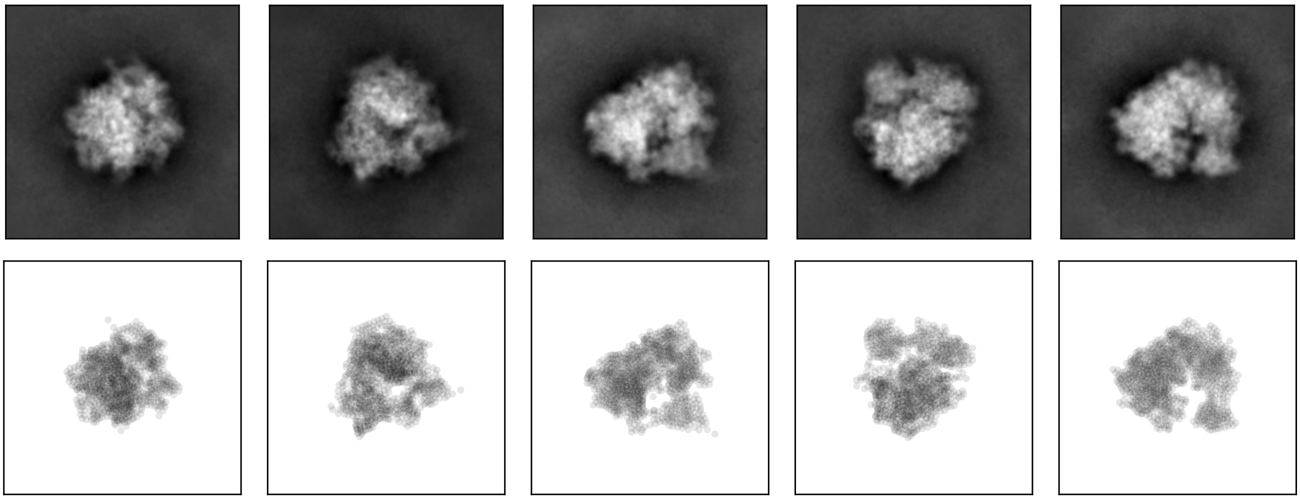

**Figure S1.** Representation of class averages by 2D point clouds. Top row: Class averages of the 80S ribosome. Bottom row: Particle representations obtained by Expectation Maximization of the central connected component.

## 2 FITTING POINT CLOUDS TO IMAGES WITH EXPECTATION MAXIMIZATION

Given a collection of pixels $\boldsymbol{u}_i$ with associated intensities $g_i$ ($i = 1, \dots, I$), we aim to represent this information by a 2D point cloud $\{\boldsymbol{y}_m; m = 1, \dots, M\}$. The model to relate the image intensities and pixels to a point cloud is a mixture of Gaussians (Eq. 12):

$$g_i \approx \alpha + \gamma \sum_{m=1}^{M} \phi_2\big(\boldsymbol{u}_i; \boldsymbol{y}_m, \sigma^2\big)$$

where $\alpha$ is a suitable background parameter, $\gamma$ is a scaling factor and $\sigma$ the width of the Gaussian components. Using the approach described in subsection 3.2, we correct for the background by masking out the intensities that are greater than a suitable threshold and subtracting the threshold from the intensity such that $\alpha = 0$. Without loss of generality we can set $\gamma = 1$. So we are trying to fit $\boldsymbol{y}_m$ and $\sigma$ such that the shifted intensities $g_i$ at pixels $\boldsymbol{u}_i$ are reproduced as closely as possible with the Gaussian mixture model:

$$g_i \approx \sum_{m=1}^{M} \phi_2\big(\boldsymbol{u}_i; \boldsymbol{y}_m, \sigma^2\big)\,.$$

This can be achieved with an Expectation Maximization (EM) algorithm that cycles over the following updates:

- Soft assignment: Each pixel $\boldsymbol{u}_i$ with associated intensity $g_i$ is assigned to a 2D point $\boldsymbol{y}_m$ with probability $p_{im}$:

$$p_{im} = \frac{\phi_2\big(\boldsymbol{u}_i; \boldsymbol{y}_m, \sigma^2\big)}{\sum_{m'=1}^{M} \phi_2\big(\boldsymbol{u}_i; \boldsymbol{y}_{m'}, \sigma^2\big)}$$

- Particle positions $\boldsymbol{y}_m$ are the centers of mass of the assigned pixels weighted by the (positive) image intensity $g_i$ and the assignment probabilities $p_{im}$ computed in the previous step:

$$\boldsymbol{y}_m = \frac{\sum_{i=1}^{I} p_{im}\, g_i \boldsymbol{u}_i}{\sum_{i=1}^{I} p_{im}\, g_i}$$

  where the denominator is the total image intensity represented by the $m$-th particle.
- The width of the Gaussians is estimated by

$$\sigma = \sqrt{\frac{1}{\sum_{i=1}^{I} g_i} \sum_{i=1}^{I} \sum_{m=1}^{M} p_{im}\, g_i\, \|\boldsymbol{u}_i - \boldsymbol{y}_m\|^2}$$

For the entire series of 400 class averages provided by SIMPLE, we fitted 1000 particles to each image. The reasoning for choosing the same number of particles is that our model predicts the same total intensity which is identical to the number of points that represent the projection images. Figure S1 shows five representative class averages of the 80S ribosome and the their point cloud representation.

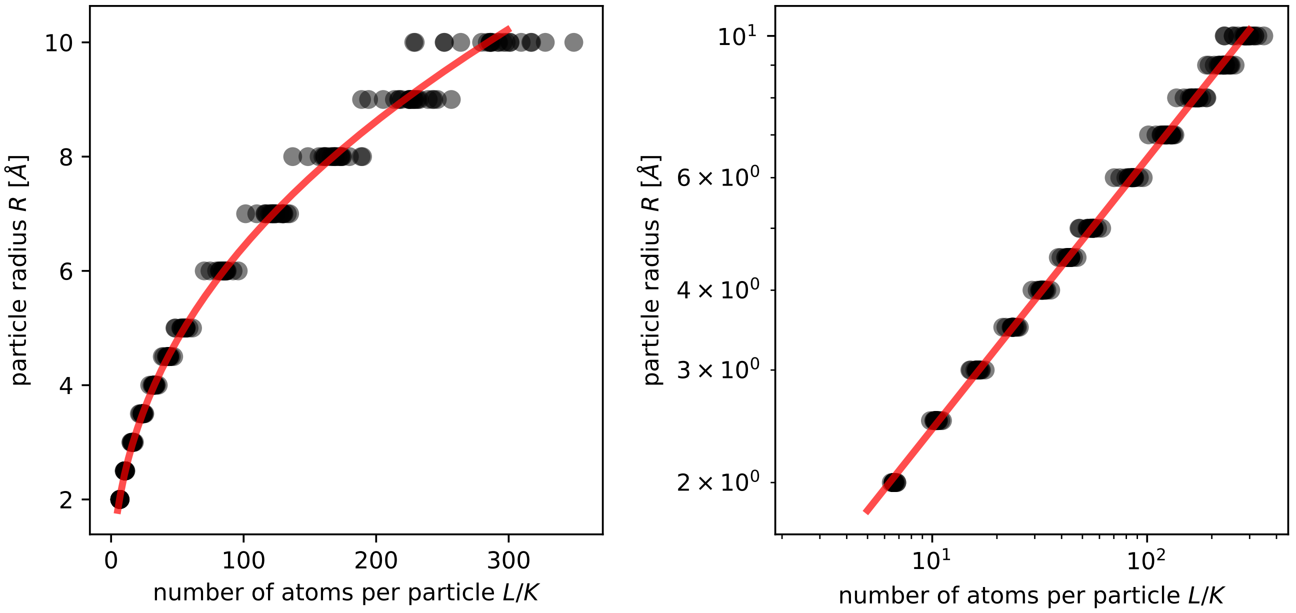


**Figure S2.** Relation between the average number of atoms per particle $(L/K)$ and the particle radius.

## 3 CHOICE OF PARAMETERS IN BOLTZMANN PRIOR

To enforce a reasonable packing of particle structures, we use a Boltzmann distribution (Eq. 17) with a repulsive pairwise distance potential (Eq. 18). The only parameters that need to be set are the particle radius $R$ and the inverse temperature $\beta$ of the Boltzmann ensemble.

### 3.1 Particle radius

Biomolecules pack in a way that is reminiscent of fluids (Liang and Dill, 2001). To design a prior distribution that favors particle configurations with similar packing characteristics, we analyzed a number of biomolecular structures at different degrees of coarse graining. The coarse-grained models were derived from atomic structures by running the DP-means algorithm (Kulis and Jordan, 2012) for different distance cutoffs $\lambda = 4, 5, 6, 7, 8, 9, 10, 12, 14, 16, 18, 20$ Å, rather than using a computationally more demanding coarse graining approach proposed by us (Chen and Habeck, 2017).

The DP-means algorithm is a variant of the K-means clustering algorithm. DP-means assigns points to cluster centers, if the Euclidean distance of a point from a center does not exceed a distance cutoff. If no cluster exists that is close enough to a given point, a new cluster is created. This process is repeated multiple times until the iterations converge. In contrast to K-means, the number of clusters varies and depends on the cutoff value $\lambda$, which is the only input parameter: smaller cutoffs result in a larger number of clusters. The clusters centers form the particle positions; the distance cutoff $\lambda$ corresponds to the particle diameter. We use the implementation of DP-means available at `https://github.com/michaelhabeck/DP-means`.

The coarse-grained structures obtained with DP-means allow us to establish a relation between the number of particles $K$ and the distance cutoff $\lambda$. In our implementation of Bayesian particle-based tomography, we work with a fixed number of particles and want to choose the particle radius $R$ such that volume-exclusion observed in known biomolecular structures is enforced. Since different biomolecular structures pack similarly, we expect that the average number of atoms per particle can be related to the particle radius

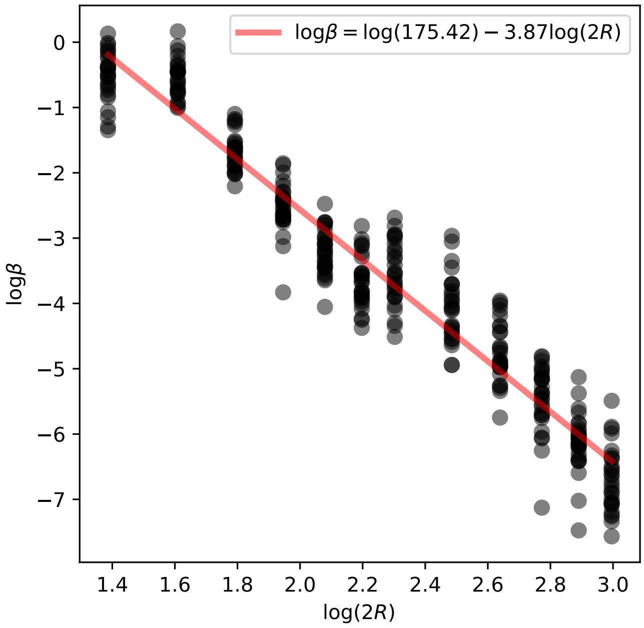


**Figure S3.** Configurational temperature as a function of particle radius.

independent of the specific structure. If $L$ denotes the total number of atoms in a biomolecular structure, then $L/K$ is the average number of atoms per particle. We use half the DP-means cutoff as a proxy for the particle radius $R = \lambda/2$. Figure S2 shows that there is indeed a high correlation between $L/K$ and $R$ that can be captured with a linear relation between the logarithms of both quantities:

$$\log R \approx -0.087 + 0.423 \log(L/K)$$

Mapping this back, we can predict a particle radius

$$R \approx 0.92 \times (L/K)^{0.42}$$

from the number of atoms $L$ and the chosen number of particles $K$. For a given biomolecular system, we compute $L$ based on the amino acid sequence.

## 3.2 Inverse temperature

The inverse temperature $\beta$ is estimated from coarse grained structures using the configurational temperature formalism (Mechelke and Habeck, 2013). For each coarse-grained model that was produced to estimate the particle radii, we computed the configurational temperature. Figure S3 shows the relation between particle diameter and the configurational temperature, which can be fitted with a straight line in log space. The inverse temperature corresponds to the slope of the line and is set to $\beta = 175$.

## 4 SAMPLING PARTICLE POSITIONS AND PRECISIONS WITH FIXED ROTATIONS

Particle positions are sampled with HMC. To test the performance of HMC, we fitted coarse-grained models of GroEL/ES against 35 simulated projection images and 2D point clouds. For both types of data, HMC generates high-quality reconstructions.

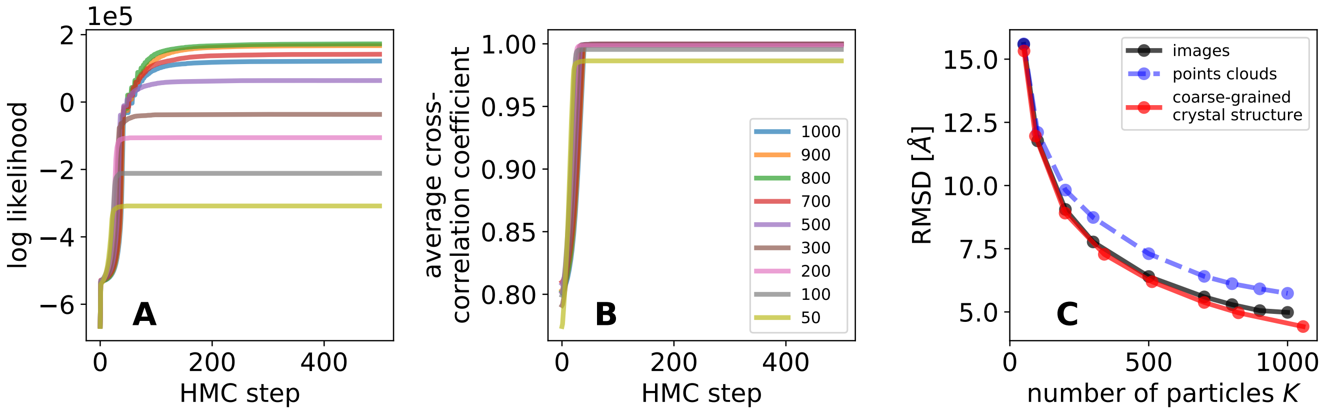


**Figure S4.** HMC sampling of particle positions with fixed rotations for a simulated class averages of GroEL/ES. **A** Evolution of the log likelihood during HMC sampling. **B** Evolution of the average cross-correlation coefficient. **C** RMSD between Carbon-alpha positions of the crystal structure and the coarse-grained models inferred with HMC. As a reference, the RMSD between the Carbon-alpha positions and the coarse-grained versions of the crystal structures is shown as red curve.

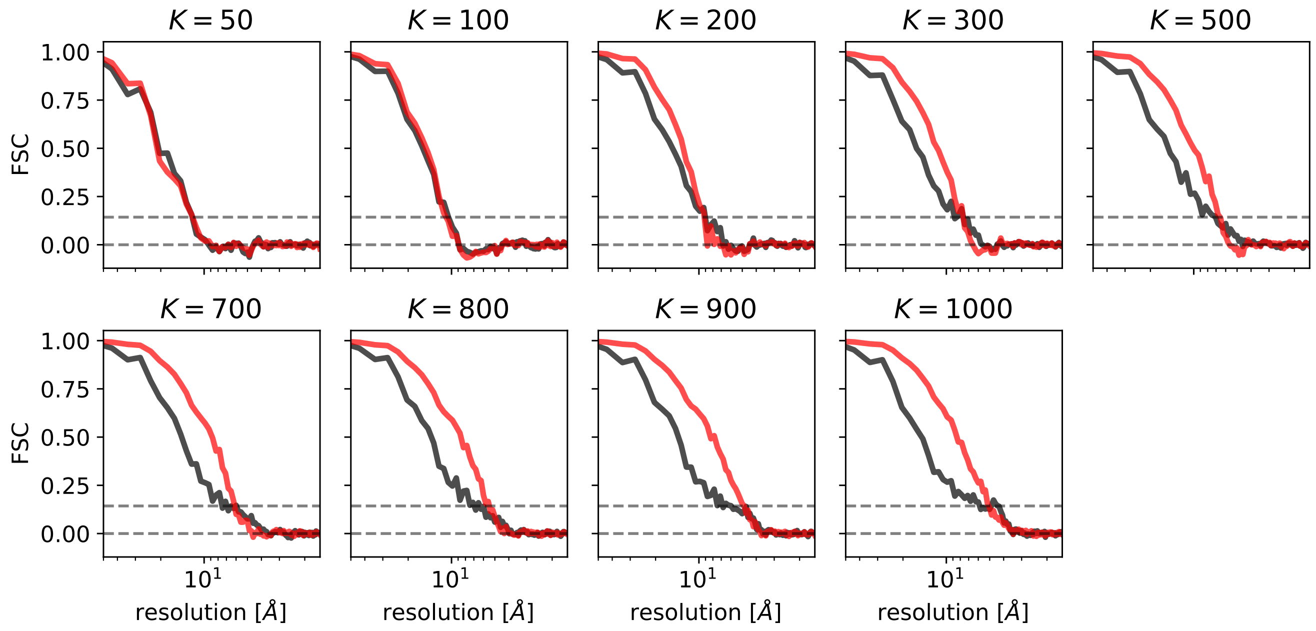


**Figure S5.** FSC curves for particle models of GroEL/ES obtained by fitting 2D point clouds (black curves) and projection images (red curves).

| **#particles** | **resolution (FSC at 0.143) [Å]** | | **RMSD [Å]** | |
|---|---|---|---|---|
| | point clouds | images | point clouds | images |
| 50 | 12.2 | 12.2 | 15.6 | 15.5 |
| 100 | 10.5 | 10.5 | 12.1 | 11.7 |
| 200 | 7.8 | 9.3 | 9.8 | 9.1 |
| 300 | 8.3 | 8.3 | 8.7 | 7.8 |
| 500 | 7.1 | 6.5 | 7.3 | 6.4 |
| 700 | 6.5 | 6.2 | 6.4 | 5.6 |
| 800 | 6.0 | 5.8 | 6.1 | 5.3 |
| 900 | 6.0 | 4.8 | 5.9 | 5.0 |
| 1000 | 5.8 | 5.0 | 5.7 | 5.0 |

**Table S1.** Resolution estimates and RMSD values for particle-based models of GroEL/ES using point clouds and projection images as input data.

# 5 POSTERIOR SAMPLING BASED ON CLASS AVERAGES OF THE 80S RIBOSOME

## 5.1 Resolution assessment

In addition to the tests based on 2D point clouds derived from the projection images, we also used the first 50 class averages themselves as an input. We assessed the accuracy of the particle models inferred from both types of input data by computing the FSC correlating the high-resolution structure EMD-2660 with the initial model generated by our method. Figure S6 shows the FSCs and Table S2 lists the resolutions derived from these curves.

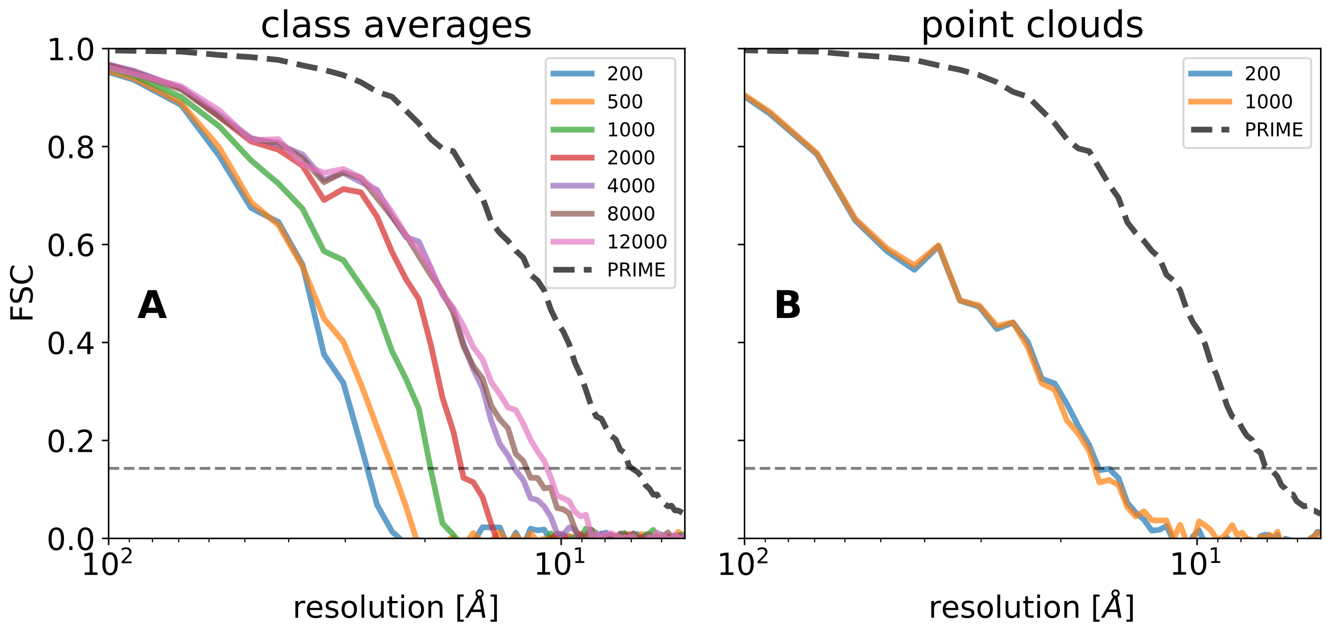


**Figure S6.** FSCs of particle models inferred from 50 class averages (**A**) and 2D points clouds (**B**).

| #particles | diameter [Å] | resolution (FSC at 0.143) [Å] |
|---|---|---|
| 200 | 32.8 | 27.6 |
| 500 | 22.3 | 23.5 |
| 1000 | 16.7 | 19.7 |
| 2000 | 12.5 | 16.9 |
| 4000 | 9.3 | 12.5 |
| 8000 | 7.0 | 11.9 |
| 12000 | 5.9 | 10.6 |

**Table S2.** Resolution estimates for particle-based models of the 80S ribosome obtained with random tomography. The FSCs are computed by comparing the high-resolution reconstruction (EMD-2660) with the density map generated from the last 100 sampled particle configurations.

## 5.2 Computation times

Computation times for 100 steps of Gibbs sampling using 50 class averages as input. Tests were run on an intel i5 processor (1.6 GHz oct-core using a single thread only).

| #particles | computation time [h] |
|---|---|
| 200 | 0.1 |
| 1000 | 0.3 |
| 4000 | 0.9 |
| 6000 | 1.2 |
| 8000 | 1.6 |
| 10000 | 2.0 |
| 12000 | 2.4 |

**Table S3.** Computation times for a 3D reconstruction from 50 class averages of the 80S ribosome using 100 Gibbs sampling steps.

## 5.3 Uncertainty Quantification

Bayesian methods and posterior sampling allow for uncertainty quantification. Since particle models can differ by a rigid transformation and a permutation of particle indices, we first need to superimpose the configurations generated by posterior sampling and establish a correspondence between particle positions across different samples. We do this by using the ICP (iterative closest point) method followed by linear assignment. After these steps, we can compute the standard deviation of each particle position which provides a structural error bar. Figure S7 shows a particle model where the error bar is encoded in the bead radius.

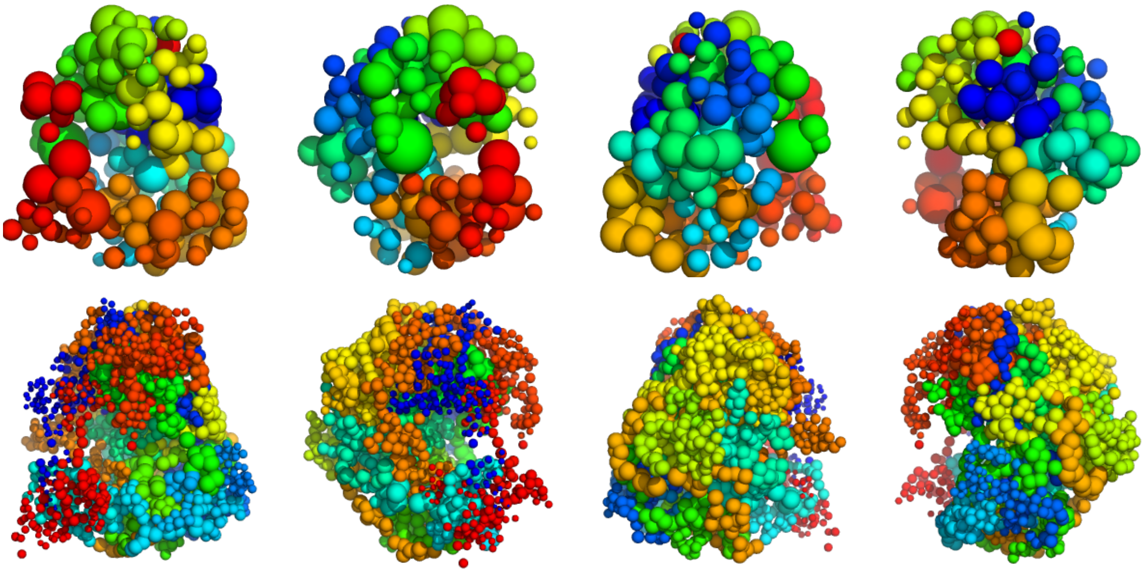

**Figure S7.** Uncertainty quantification of the ribosome model. The size of the spheres is proportional to the standard deviation of particle positions after superposition and assignment. The top row shows a model based on 200 particles. The bottom row shows the structure based on 2000 particles. Shown are side views of the 80S ribosome that differ by a 90-degree rotation about the $z$ axis.

# 6 POSTERIOR SAMPLING BASED ON CLASS AVERAGES OF BETA-GALACTOSIDASE

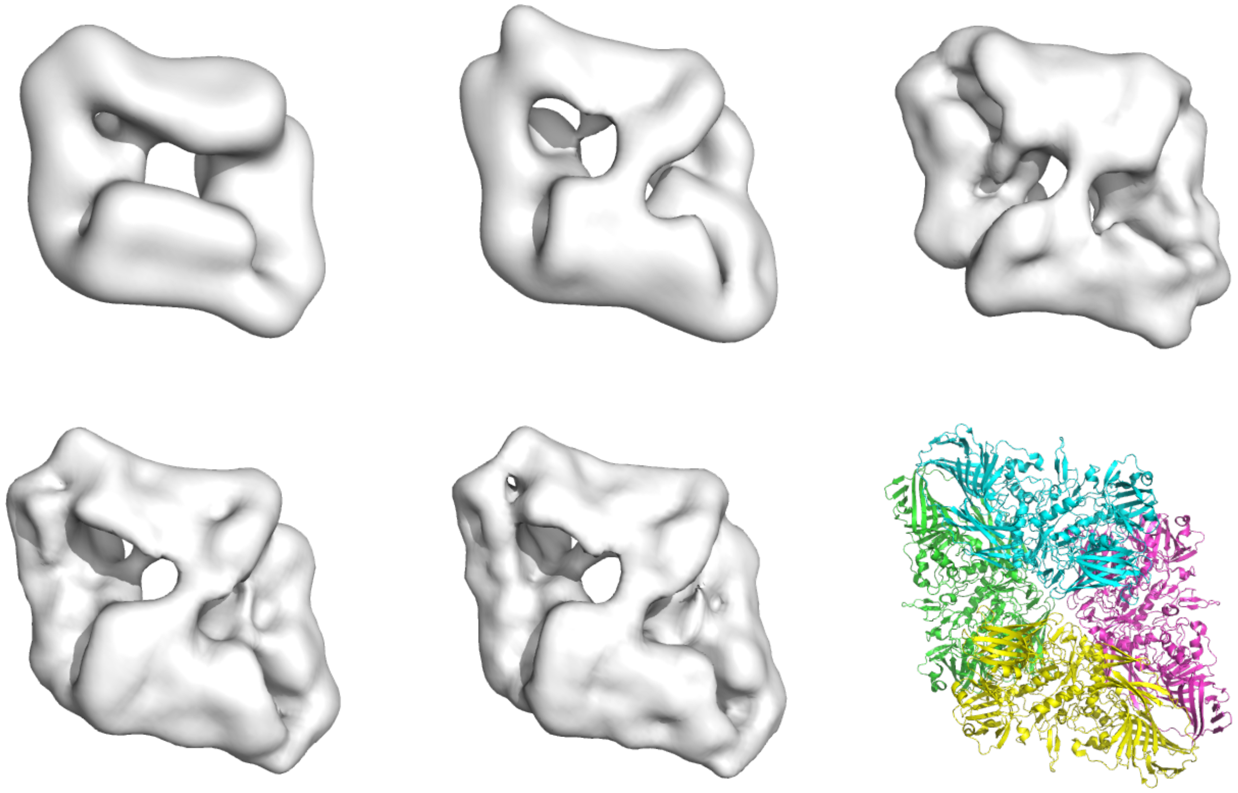

**Figure S8.** Density maps of beta-galactosidase obtained with Bayesian random tomography using 16 class averages as input. Top row: 100, 200, 500 (left to right), Bottom row: 1000, 2000, PDB code 1jz8 (left to right).

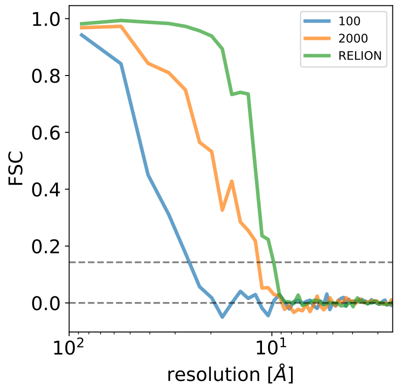


**Figure S9.** FSC curves comparing the high-resolution reconstruction EMD-5995 with Bayesian models using 100 and 2000 particles, respectively, as well as an initial structure obtained with RELION.

## 7 IMPACT OF BOLTZMANN PRIOR

To assess the impact of the Boltzmann prior (Eq. 17), we ran simulations with the beta-galactosidase images where the Boltzmann prior was switched on and off ($\beta = 0$).

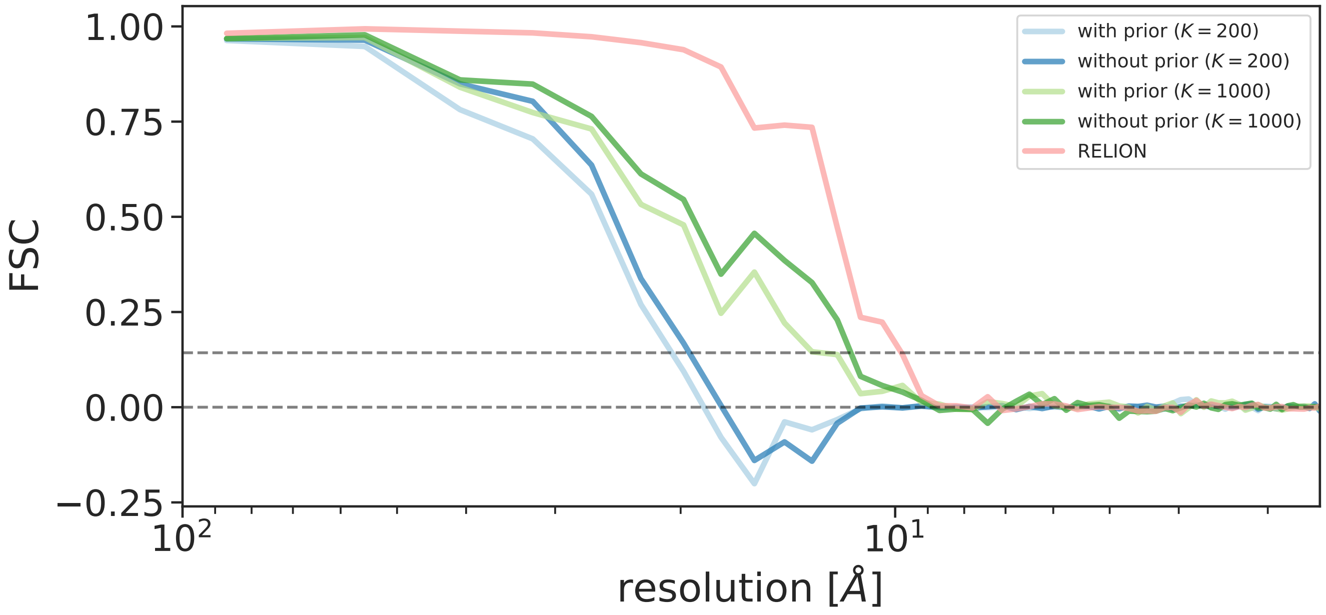


**Figure S10.** Impact of Boltzmann prior on the accuracy of the 3D reconstruction. FSC between high-resolution reconstruction EMD-5995 and models calculated with and without Boltzmann prior for various numbers of particles. The FSC curve for the RELION's initial model is shown in yellow.

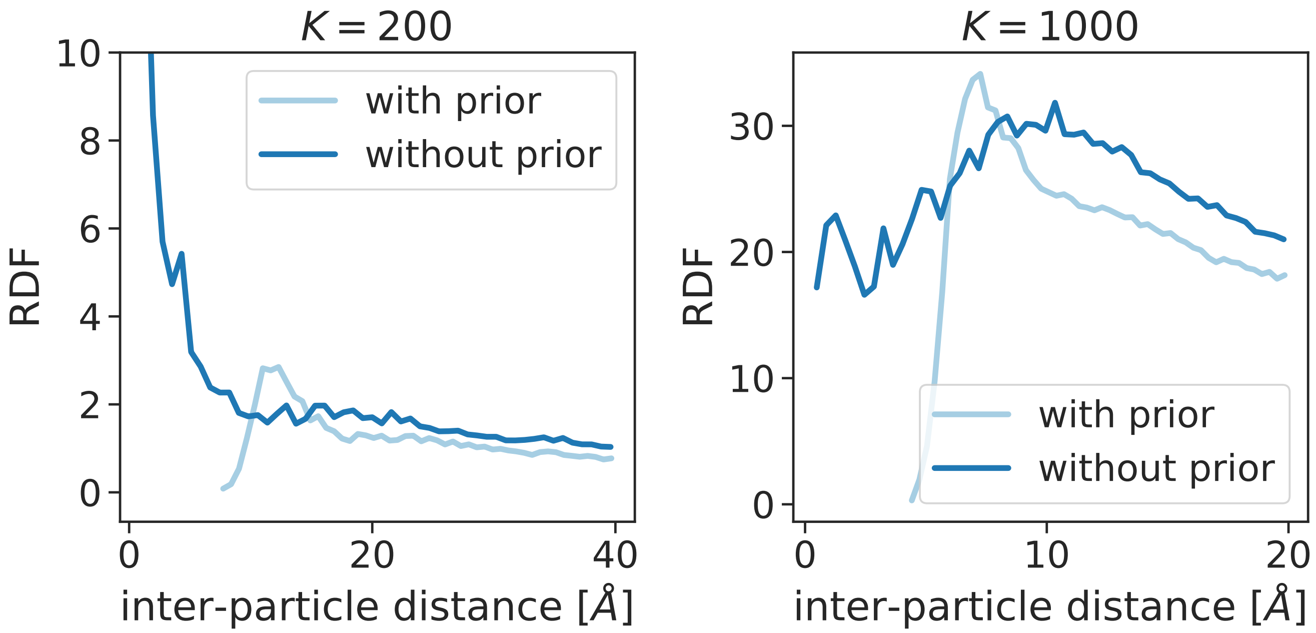


**Figure S11.** Impact of Boltzmann prior on particle packing. Shown are the radial distribution functions computed from sampled particle models of beta-galactosidase with (light blue line) and without Boltzmann prior (dark blue line).

## REFERENCES

Liang J, Dill KA. Are proteins well-packed? *Biophys. J.* **81** (2001) 751–766.

Kulis B, Jordan MI. Revisiting k-means: New algorithms via bayesian nonparametrics. Langford J, Pineau J, editors, *Proceedings of the 29th International Conference on Machine Learning (ICML-12)* (New York, NY, USA) (2012), 513–520.

Chen YL, Habeck M. Data-driven coarse graining of large biomolecular structures. *PLoS ONE* **12** (2017) e0183057.

Mechelke M, Habeck M. Estimation of Interaction Potentials through the Configurational Temperature Formalism. *J Chem Theory Comput* **9** (2013) 5685–5692. doi:10.1021/ct400580p.
